# Severity and mortality of severe *Plasmodium ovale* infection: A systematic review and meta-analysis

**DOI:** 10.1371/journal.pone.0235014

**Published:** 2020-06-19

**Authors:** Manas Kotepui, Kwuntida Uthaisar Kotepui, Giovanni D. Milanez, Frederick R. Masangkay

**Affiliations:** 1 Medical Technology, School of Allied Health Sciences, Walailak University, Nakhon Si Thammarat, Thailand; 2 Department of Medical Technology, Institute of Arts and Sciences, Far Eastern University-Manila, Manila, Philippines; Instituto Rene Rachou, BRAZIL

## Abstract

*Plasmodium ovale* can infect humans, causing malaria disease. We aimed to investigate the severity and mortality of severe *P*. *ovale* infection to increase the awareness of physicians regarding the prognosis of this severe disease and outcome-related deaths in countries in which this disease is endemic. Articles that were published in the PubMed, Scopus, and ISI Web of Science databases prior to January 5, 2020 and reported the prevalence of severe *P*. *ovale* infection were systematically searched and reviewed. Studies that mainly reported severe *P*. *ovale* infection according to the 2014 WHO criteria for the treatment of malaria were included. Two reviewers selected, identified, assessed, and extracted data from studies independently. The pooled prevalence of severe *P*. *ovale* mono-infections was estimated using the command “metaprop case population, random/fixed”, which yielded the pooled estimate, 95% confidence interval (CI) and the I^2^ value, indicating the level of heterogeneity. Meta-analyses of the proportions were performed using a random-effects model to explore the different proportions of severity between patients with *P*. *ovale* and those with other *Plasmodium* species infections. Among the eight studies that were included and had a total of 1,365 ovale malaria cases, the pooled prevalence of severe *P*. *ovale* was 0.03 (95% CI = 0.03–0.05%, I^2^ = 54.4%). Jaundice (1.1%), severe anemia (0.88%), and pulmonary impairments (0.59%) were the most common severe complications found in patients infected with *P*. *ovale*. The meta-analysis demonstrated that a smaller proportion of patients with *P*. *ovale* than of patients with *P*. *falciparum* had severe infections (P-value = 0.01, OR = 0.36, 95% CI = 0.16–0.81, I^2^ = 72%). The mortality rate of severe *P*. *ovale* infections was 0.15% (2/1,365 cases). Although severe complications of *P*. *ovale* infections in patients are rare, it is very important to increase the awareness of physicians regarding the prognosis of severe *P*. *ovale* infections in patients, especially in a high-risk population.

## Introduction

Severe malaria results in the dysfunction of one or more vital organs [1]. *Plasmodium ovale*, which causes tertian malaria, was first reported in 1922 as one of the five *Plasmodium* species that can infect humans [2]. *P*. *ovale* accounts for between 0.5 and 10.5% of all malaria cases, and it is geographically distributed in sub-Saharan Africa, the Western Pacific, Timor, and Indonesia [3]. The highest prevalence of *P*. *ovale* has been reported in Papua New Guinea (15%) [4] and Nigeria (15%) [5]. However, the most recent retrospective cohort study conducted in Papua, Indonesia during 2004–2013 demonstrated a low prevalence of *P*. *ovale* infections (0.06%) among 68,361 patients [6]. Other recent studies demonstrated that *P*. *ovale* infections accounted for 2.5% of malaria cases in Uganda [7] and 2.7% of malaria cases in China [8]. Infections due to *P*. *ovale* have been underestimated compared with those of other *Plasmodium* species, as *P*. *ovale* has been demonstrated to lead to low parasitemia and have morphologic similarities with *P*. *vivax* and mixed infections [9, 10]. Similar to *P*. *vivax*, *P*. *ovale* can cause relapsing infection due to the presence of latent parasites (hypnozoites) in the liver long after the first treatment is administered with anti-malarial drugs [3]. *P*. *ovale* is detected and identified with the standard microscopic method. Rapid diagnostic tests (RDTs) can also be used in cases where microscopic detection cannot be performed, such as in rural or remote areas. However, the sensitivity of RDTs to detect *P*. *ovale* is low (22.2%) [11]. The low sensitivity of RDTs can be attributed to the low parasitemia level [12, 13] or different targeted antigens [14]. Recently, a molecular method with nested polymerase chain reaction (nested PCR) has been used to identify *Plasmodium* species [15]. Although continuous efforts regarding PCR techniques have been made to develop new diagnostic techniques, such as *Plasmodium* species-specific PCR-restriction fragment length polymorphism (PCR-RFLP) [16], for identifying malaria parasites, nested PCR techniques with high sensitivity and specificity are needed.

According to the molecular technique, two subspecies of *P*. *ovale*, *curtisi* and *P*. *ovale wallikeri*, were identified in 2010 by a nested PCR detection assay of dimorphism in the gene encoding the *P*. *ovale* tryptophan-rich antigen (potra) in West Africa [17]. Currently, *P*. *falciparum* is still the leading cause of severe malaria [18]. *P*. *ovale* is usually associated with low morbidity and mortality. However, *P*. *ovale* can cause severe complications and death [19, 20]. Previous studies have reported that the severe complications of *P*. *ovale* infections include acute respiratory distress syndrome (ARDS) [21–26], renal impairment [24, 27], jaundice, and hypotension [26, 27]. The most recent study on severe *P*. *ovale* malaria in travelers and migrants demonstrated that 5.3% of patients with *P*. *ovale* developed severe complications according to the 2015 WHO criteria, including hyperbilirubinemia, pulmonary edema, shock, significant bleeding, and impaired consciousness [28]. There are a limited number of systematic reviews and meta-analyses on severe *P*. *ovale* malaria. A previous systematic review of 33 articles published between 1922 and 2015 demonstrated that a total of five out of 22 severe cases of *P*. *ovale* malaria were fatal, and two cases of congenital *P*. *ovale* malaria were fatal [29]. A more recent systematic review conducted by Yerlikaya et al. in 2018 demonstrated that a RDT had poor performance in detecting *P*. *ovale* because *P*. *ovale* infections usually occur at very low parasite densities, leading to missed detection by microscopy and RDTs [30]. Nevertheless, studies on the prevalence of severe *P*. *ovale* malaria provide more information on this neglected species and are urgently needed. This systematic review and meta-analysis aimed to investigate the severity and mortality rates of severe *P*. *ovale* infection to increase the awareness of physicians regarding the prognosis of this severe disease and outcome-related deaths in countries in which this disease is endemic and to identify the differences in the proportions of patients with severe *P*. *ovale* manifestations and with other severe *Plasmodium* spp. infections.

## Methods

### Study selection

This systematic review was designed on the basis of the Preferred Reporting Items for Systematic Reviews and Meta-Analyses (PRISMA) guidelines (S1 Checklist). Two authors (MK and KUK) searched the Medline, Scopus, and ISI Web of Science databases independently for articles published in English prior to January 5, 2020. Additional articles from other databases were also selected. The search terms, which included the Boolean operators “OR” or “AND”, were as follows: “(severe OR complicated OR complication) AND (Plasmodium ovale)” (S1 Table). EndNote software version X7 (Thomson Reuters, New York, NY) was used to process all references in our study.

### Quality of the included studies

The quality of the observational studies was assessed in accordance with the Newcastle-Ottawa Scale (NOS) [31]. A 'star system', in which a study is judged on the basis of the following three broad perspectives was used: the selection of the study groups; the comparability of the groups; and the ascertainment of either the exposure or outcome of interest.

### Data extraction

All raw and available data from eight studies were extracted to explore the prevalence of severe complications in patients with *P*. *ovale* infections. Two authors (MK and KUK) extracted the data from the selected studies. If there were any discrepancies between the two reviewers, another reviewer (GM) determined whether the study should be included. For the prevalence outcome of severe *P*. *ovale* malaria, all prospective cohort, cross-sectional studies, and case-control studies that reported the number of patients with severe complications from *P*. *ovale* were included. The following articles were excluded from our study: book and book chapter reviews, conference papers, editorials, letters, correspondences, notes, reviews, animal studies, case reports, drug studies and clinical trials, entomological studies, experimental studies, knowledge assessments, studies written in local languages, studies on mixed infections of *P*. *ovale* and other *Plasmodium* spp., studies of *P*. *ovale* during pregnancy, prevalence studies of non-*P*. *ovale* infections, and prevalence studies of *P*. *ovale* that did not report data on severe complications.

### Statistical analysis

The primary outcome of the present study was the pooled prevalence of severe *P*. *ovale* infections and mortality rates. The number of severe *P*. *ovale* infections and the total number of *P*. *ovale* infections were entered into an Excel data sheet (Microsoft Corporation, USA). The pooled prevalence of the severity of *P*. *ovale* mono-infections was estimated using the command “metaprop case population, random/fixed” available in STATA software version 15.0 (‎StataCorp LLC, USA). The results are presented as the pooled estimate and 95% confidence interval (CI). A meta-regression with the median age as a covariate was performed to evaluate whether the age of the participants was a confounder of the pooled prevalence of severe *P*. *ovale*.

The secondary outcome of the present study was the different proportions of severity between patients with *P*. *ovale* and those with other *Plasmodium* species. The meta-analyses of the proportions were performed using a random-effects model provided in Review Manager 5.3 software (Cochrane Community). The heterogeneity was assessed with the Mantel-Haenszel method, and the I^2^ values were also calculated. The I^2^ was considered low (<25%), moderate (25–50%), or high (>50%). A fixed-effects model was used when I^2^<50%, whereas a random-effects model was used when I^2^>50%. The publication bias was also assessed using funnel plots.

## Results

### Characteristics of included studies

**Fig 1:**
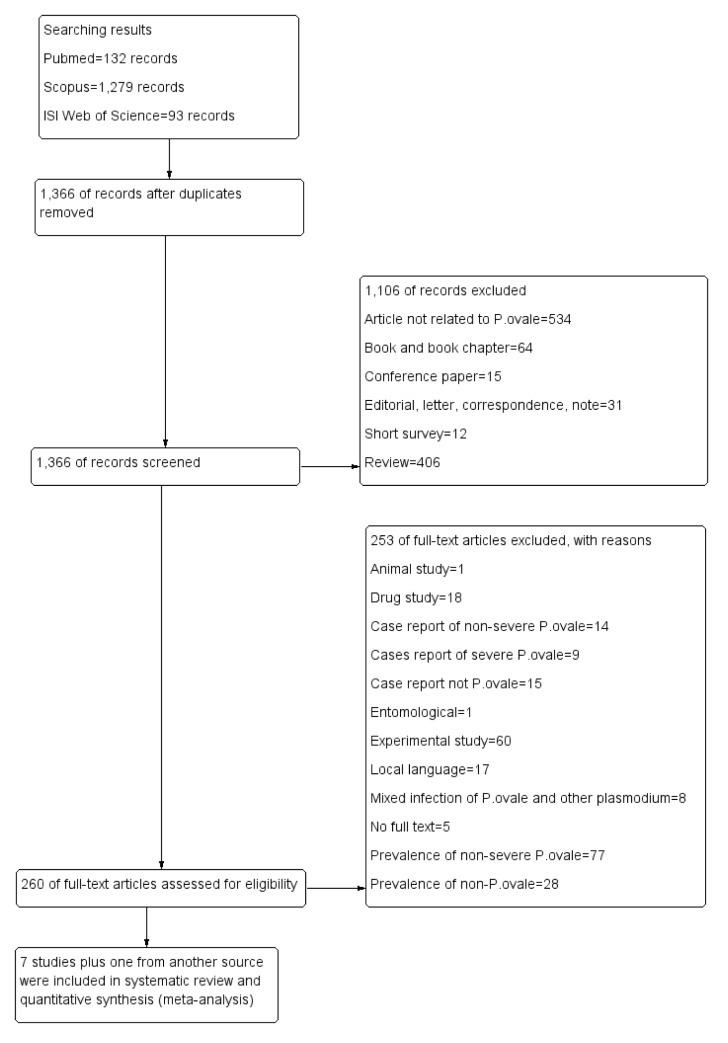
Flow diagram. Flow chart of the study selection process.

A total of 1,504 articles were retrieved from three databases during the search, and eight of these articles, including retrospective studies, prospective studies, and case series, were included in this study (Fig 1). The characteristics of the eight studies are shown in Table 1. The studies were conducted in Belgium (2000–2005) [32], the Ivory Coast (2007–2008) [33], the US National Malaria Surveillance System (NMSS) (1985–2011) [19], Indonesia (2004–2013) [34], Ethiopia (2013–2014) [35], Italy (2014–2017) [36], Spain (2005–2011) [37], and Sweden (1995–2015) [38]. The ages of the participants were reported in seven of the included studies [32–38], as they were not reported in a study by Hwang et al. [19]. Five studies reported severe imported *P*. *ovale* infections in European countries, including Belgium [32], France [33], Italy [36], Spain [37], and Sweden [38], while other studies reported severe *P*. *ovale* infections in the United States of America [19], Indonesia [34], and Ethiopia [35]. Most of the included studies (7/8, 87.5%) used microscopy as the gold standard for malaria identification, except for a study conducted by Ramos et al. in 2016 [35]. The combination of microscopy and PCR was used in three of the included studies [19, 36, 37]. Most of the *Plasmodium* spp. infections that were reported among the included studies were caused by *P*. *falciparum* (116,898 cases, 51%), *P*. *vivax* (78,282 cases, 34.1%), mixed infection (26,049 cases, 11.4%), *P*. *malariae* (6,428 cases, 2.8%), and *P*. *ovale* (1,365 cases, 0.6%).

**Table 1:** Characteristics of the included studies.

No.	Reference	Study area (years of the survey)	Participants	Age range	Reference method for malaria identification	*Plasmodium* sp.	Severe malaria	Fetal	Impaired consciousness	Prostration	Severe anemia	Renal impairment	Hyperbilirubinemia	Pulmonary	Significant bleeding	Shock
1.	Bottieau et al., 2006	Belgium (2000–2005)	Travelers, expatriates, and foreign Visitors	35 (11–77)	Microscopy	Pv = 48	Pv = 8						Pv = 8			
						Po = 34	Po = 4						Po = 4			
						Pm = 16	Pm = 3						Pm = 3			
2	de Laval et al., 2010	France (2007–2008)	French soldiers	32 (30–36)	Microscopy	Case series	Po = 1						Po = 1			
						Po = 6										
3.	Hwang et al., 2014	USA (1985–2011)	Travelers	NA	Microscopy, PCR	Pf = 15,272	Pf = 1,416	Pf = 122	Pf = 514		Pf = 140	Pf = 503		Pf = 176		
						Pv = 12,152	Pv = 163	Pv = 10	Pv = 37		Pv = 16	Pv = 48		Pv = 35		
						Po = 903	Po = 18	Po = 2	Po = 4		Po = 6	Po = 6		Po = 4		
						Pm = 1254	Pm = 22	Pm = 2	Pm = 2		Pm = 3	Pm = 7		Pm = 2		
						Mixed = 226	Mixed = NA									
4.	Langford et al., 2015	Indonesia (2004–2013)	Patients presenting to the hospital	<1	Microscopy	Pf = 100,078	Pf = 4,031				Pf = 2,521	Pf = 415		Pf = 1,095		
				1-<5		Pv = 65,306	Pv = 2,118				Pv = 1,099	Pv = 81		Pv = 938		
				5-<15							Po = 0	Po = 0		Po = 1		
				≥ 15		Po = 120	Po = 1				Pm = 100	Pm = 16		Pm = 44		
						Pm = 5,097	Pm = 160				Mixed = 782	Mixed = 84		Mixed = 343		
						Mixed = 25,779	Mixed = 1,209									
5.	Ramos et al., 2016	Ethiopia (2013–2014)	Patients with severe anemia	15 (0.5–65)	PCR	111	Pf = 18				Pf = 18					
							Pv = 4				Pv = 4					
							Po = 4				Po = 4					
6.	Rojo-Marcos et al., 2018	Italy (2014–2017)	Patients with imported *P*. *ovale*	35 (22.2–53)	Microscopy, PCR	79	Po = 5				Po = 1		Po = 4			
7.	Rojo-Marcos et al., 2014	Spain (2005–2011)	Patients with imported *P*. *ovale*	36.5 (11.8–52.7)	Microscopy, PCR	35	Po = 3				Po = 2			Po = 1		
8.	Wangdahl et al., 2019	Sweden (1995–2015)	Travelers and Migrants	All = 32.6 (0.2–83)	Microscopy	Pf = 1,548	Pf = 146	Pf = 3	Pf = 28	Pf = 9	Pf = 16	Pf = 31	Pf = 66	Pf = 15	Pf = 17	Pf = 33
						Pv = 776	Pv = 60	Pv = 0	Pv = 1	Pv = 1	Pv = 15	Pv = 1	Pv = 28	Pv = 4	Pv = 5	Pv = 8
				Pf = 34.4 (0.2–83)		Po = 188	Po = 10	Po = 0	Po = 1	Po = 0	Po = 0	Po = 0	Po = 6	Po = 2	Po = 1	Po = 2
						Pm = 61	Pm = 2	Pm = 0	Pm = 0	Pm = 0	Pm = 1	Pm = 0	Pm = 0	Pm = 0	Pm = 0	Pm = 1
				Pv = 29.9 (1–79)		Mixed = 44	Mixed = 8	Mixed = 1	Mixed = 1	Mixed = 0	Mixed = 4	Mixed = 3	Mixed = 4	Mixed = 0	Mixed = 1	Mixed = 3
				Pm = 30.2 (3–65)												
				Po = 30.2 (3–65)												
		Total cases				Pf = 116,898	Pf = 5,611	Pf = 125	Pf = 542	Pf = 9	Pf = 395	Pf = 949	Pf = 66	Pf = 1,286	Pf = 17	Pf = 33
						Pv = 78,282	Pv = 2,353	Pv = 10	Pv = 38	Pv = 1	Pv = 1,134	Pv = 130	Pv = 36	Pv = 977	Pv = 5	Pv = 8
						Po = 1,365	Po = 46	Po = 2	Po = 5	Po = 2	Po = 13	Po = 6	Po = 15	Po = 8	Po = 1	Po = 2
						Pm = 6,428	Pm = 187	Pm = 2	Pm = 2	Pm = 2	Pm = 104	Pm = 23	Pm = 3	Pm = 46	Pm = 0	Pm = 1
						Mixed = 26,049	Mixed = 1,217	Mixed = 1	Mixed = 1	Mixed = 1	Mixed = 786	Mixed = 87	Mixed = 4	Mixed = 343	Mixed = 1	Mixed = 3

### Prevalence of severe *P*. *ovale* infections

**Fig 2:**
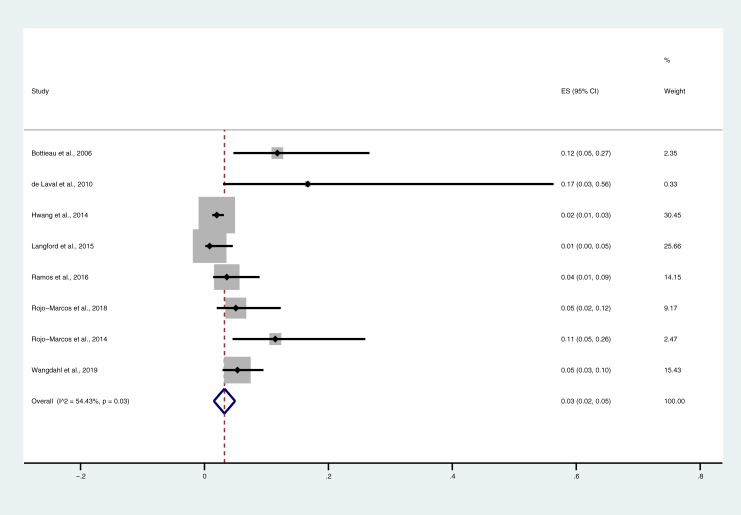
Pooled prevalence of severe *P*. *ovale* infections. Forest plot comparing the proportions of severe *P*. *ovale* cases and *P*. *vivax* cases.

Among the eight included studies, which included a total of 1,365 ovale malaria cases, the pooled prevalence of severe *P*. *ovale* was 0.03 (95% CI = 0.03–0.05%, I^2^ = 54.4%). The highest proportions of severe *P*. *ovale* were found in the study by de Laval et al. [33] (OR = 0.17, 95% CI = 0.03–0.56) and the study by Bottieau et al. [32] (OR = 0.12, 95% CI = 0.05–0.27), whereas the lowest proportion was found in the study by Langford et al., 2015 [34] (Fig 2). According to the results of the meta-regression of six included studies, the age of the participants did not confound to the pooled prevalence of severe *P*. *ovale* infections (P-value = 0.3, 95% CI = -0.002–0.005). The complications most commonly found in patients with *P*. *ovale* infections were jaundice (1.1%), severe anemia (0.88%), and pulmonary impairments (0.59%) (Table 2).

**Table 2:** Complications associated with *P*. *ovale*.

Major complication (WHO, 2014)	Total number of patients with a severe case	Proportion of complications (%) among the total number of *P*. *ovale* cases (1,365 cases)
Pulmonary impairment	8	0.59
Cerebral malaria	5	0.37
Renal impairment	6	0.44
Prostration	2	0.15
Hypotension/shock	2	0.15
Jaundice	15	1.10
Severe anemia	13	0.95
Bleeding/DIC	1	0.07

### Comparison of severity between the *P*. *ovale* and *Plasmodium* spp. infections

**Fig 3:**
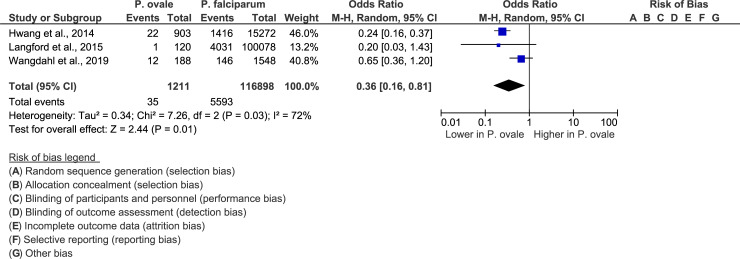
Forest plot comparing severe *P*. *ovale* cases and *P*. *falciparum* cases. The forest plot compared the proportions of patients with severe *P*. *ovale* infections with that of patients with *P*. *falciparum* infections.

The meta-analysis of three included studies [19, 34, 38] demonstrated that a smaller proportion of patients with *P*. *ovale* than of patients with *P*. *falciparum* had severe infections (P-value = 0.01, OR = 0.36, 95% CI = 0.16–0.81, I^2^ = 72%) (Fig 3).

**Fig 4:**
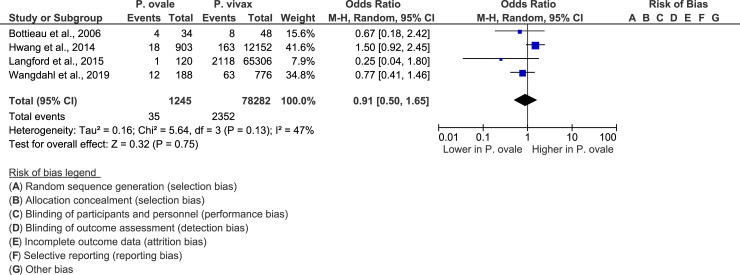
Forest plot comparing severe *P*. *ovale* cases and *P*. *vivax* cases. The forest plot compared the proportions of severe *P*. *ovale* cases and *P*. *vivax* cases.

The meta-analysis of four studies [19, 32, 34, 38] revealed there are no significant differences in the proportion of patients with severe *P*. *ovale* infections and that of patients with severe *P*. *vivax* infections (P-value = 0.75, OR = 0.91, 95% CI = 0.5–1.65, I^2^ = 47%) (Fig 4).

**Fig 5:**
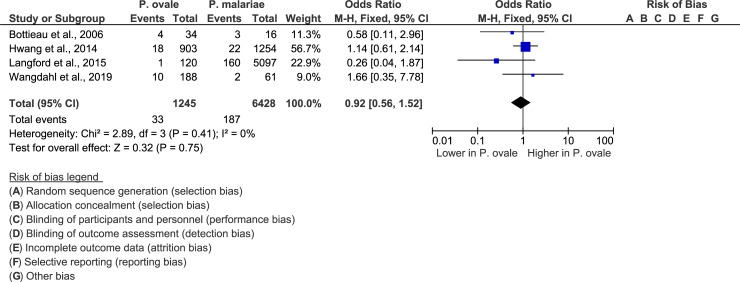
Forest plot comparing severe *P*. *ovale* cases and *P*. *malariae* cases. Forest plot comparing the proportion of patients with severe *P*. *ovale* cases and the proportion of patients with *P*. *malaria* cases.

The meta-analysis of four studies [19, 32, 34, 38] demonstrated that there was no significant difference between the proportion of patients with severe *P*. *ovale* infections and the proportion of patients with severe *P*. *malariae* infections (P-value = 0.75, OR = 0.92, 95% CI = 0.56–1.52, I^2^ = 0%) (Fig 5).

### Mortality rates of *P*. *ovale* and *Plasmodium* spp. infections

There was only one study that reported death cases resulting from *P*. *ovale* infections [19].

The mortality rate of severe *P*. *ovale* infections in their study was 0.22% (2/903). The mortality rate of severe *P*. *ovale* infections among the eight included studies was 0.15% (2/1,365 cases). The mortality rate of other *Plasmodium* infections reported in the eight included studies was 0.11% for *P*. *falciparum* infections, 0.013% for *P*. *vivax* infections, 0.03% for *P*. *malariae* infections, and 0.004% for mixed infections.

### Quality of included studies

Four studies were rated as having 9 stars (good quality), three studies were rated as having 7 stars (adequate quality), and one study was rated as having 6 stars (adequate quality) according to the NOS concerning the selection process for the cases and controls included (Table 3).

**Table 3:** Quality of the included studies.

No.	Reference	Selection				Compatibility	Exposure		
		Is the Case Definition Adequate?	Representativeness of the Cases	Selection of Controls	Definition of Controls		Ascertainment of Exposure	Same method of ascertainment for cases and controls	Non-Response Rate
1.	Bottieau et al., 2006	**✵**	**✵**	**✵**	**✵**	**✵✵**	**✵**	**✵**	****✵****
2	de Laval et al., 2010	**✵**	**✵**			**✵✵**	**✵**	**✵**	**✵**
3.	Hwang et al., 2014	**✵**	**✵**	**✵**	**✵**	**✵✵**	**✵**	**✵**	**✵**
4.	Langford et al., 2015	**✵**	**✵**	**✵**	**✵**	**✵✵**	**✵**	**✵**	**✵**
5.	Ramos et al., 2016	**✵**				**✵✵**	**✵**	**✵**	**✵**
6.	Rojo-Marcos et al., 2018	**✵**	**✵**			**✵✵**	**✵**	**✵**	**✵**
7.	Rojo-Marcos et al., 2014	**✵**	**✵**			**✵✵**	**✵**	**✵**	**✵**
8.	Wangdahl et al., 2019	**✵**	**✵**	**✵**	**✵**	**✵✵**	**✵**	**✵**	**✵**

### Publication bias

A funnel plot analysis of the included studies could not be performed to assess the publication bias among the included studies, as a minimum of 10 studies were required for the analysis [39].

## Discussion

The present systematic review and meta-analysis aimed to explore the prevalence of severe *P*. *ovale* infections and to summarize the mortality caused by *P*. *ovale*. The results demonstrated that 3% of patients infected with *P*. *ovale* developed severe complications according to the WHO 2014 guidelines [1]. Therefore, the term “benign malaria” might not apply to patients tested positive with *P*. *ovale*, especially travelers after returning from countries in which *P*. *ovale* is endemic. In addition, international and national healthcare providers should be recommending the use of chemoprophylaxis for travelers, as the present study revealed that *P*. *ovale*, long-recognized as benign malaria, can cause severe and fatal clinical manifestations. In the present study, a meta-regression with age as a covariate was performed, and the pooled prevalence of severe *P*. *ovale* infections was investigated. The results showed that the age of the participants in six of the included studies did not confound the pooled prevalence of severe *P*. *ovale* infections. A previous study demonstrated that the highest incidence of *P*. *ovale* was observed among children 0–7 years old, who had an 8-fold higher risk of *P*. *ovale* infections than did adults; nevertheless, clinical attacks were observed in all age groups [40].

The present study also demonstrated a significant difference between the proportion of patients with severe *P*. *ovale* infections and severe *P*. *falciparum* infections. A lower proportion of patients with severe *P*. *ovale* infections than of patients with *P*. *falciparum* infections had severe cases. This finding indicated that although *P*. *ovale* can cause severe malaria, the chance of patients developing severe complications is lower with *P*. *ovale* than with *P*. *falciparum*, which is the *Plasmodium* species widely known to be the leading cause of severe malaria in humans. This study also demonstrated there are no significant differences in the proportions of patients with severe *P*. *ovale* and with *P*. *vivax*/*P*. *malariae*. This finding demonstrated that there are relatively few severe *P*. *ovale* cases, including *P*. *vivax* and *P*. *ovale* cases. In contrast to *P*. *vivax*, which is endemic in Asia, Central America, and South America, *P*. *ovale* is only endemic in some African countries [3]. However, the study conducted by Langford et al. in 2015 [34] indicated that there were 120 cases of *P*. *ovale* infection in Indonesia, whereas other included studies reported that travelers who returned to their home countries had imported *P*. *ovale* malaria cases [19, 32, 35–38].

Although microscopy is still considered to be the gold standard for the identification of *Plasmodium* species, the morphological characteristics of *P*. *ovale* are similar to those of *P*. *vivax* and are readily missed by less experienced microscopists [41, 42]. This problem may have caused certain *P*. *ovale* cases to have been missed in the included studies conducted by Bottieau et al. [32], de Laval et al. [33], Langford et al. [34], and Wangdahl et al. [38], as only microscopy was used to identify malaria parasites. Missed *P*. *ovale* cases have been reported when only microscopy was used [43]. In addition, missed diagnoses of *P*. *ovale* infections leading to relapses with a low parasitemia level is a problem related to this *Plasmodium* species [44]. Previous studies have indicated that the sensitivity of routine microscopy in detecting *P*. *ovale* in imported cases is very low and related to low parasitemia levels [32, 45]. Moreover, the rapid diagnosis tests (RDTs) that have been developed and are available lack the sensitivity required to detect low amounts of the circulating antigen of *P*. *ovale* [46, 47]. Moreover, a previous study indicated that routine microscopic examinations with thick and thin blood smears should be repeated three times in cases of suspected imported *P*. *ovale* malaria [48]. Ideally, molecular detection using polymerase chain reaction (PCR) to detect and confirm *P*. *ovale* infections in cases of low parasitemia or negative blood film is needed, although it is not routinely available [32, 43]. Therefore, the limitations of routine microscopic and RDT tests might affect the management/treatment of patients and lead to an increase in the morbidity associated with *P*. *ovale* infections [49]. The mortality caused by severe *P*. *ovale* infections among travelers who returned to the USA was previously reported by Hwang et al. in 2014 [19]. The authors recommended the use of suppressive prophylaxis and antirelapse treatment with primaquine drugs for patients who returned from ovale-endemic countries [19]. Nevertheless, a previous case report showed that severe complications led to fatality in patients who had received anti-malarial prophylaxis treatment during their trip to Nigeria [20]. This finding may have been caused by the impropriate usage of prophylactic drugs or the survival of hypnozoites during anti-malaria chemoprophylaxis against the *P*. *ovale* infection [50].

The mechanism by which *P*. *ovale* infections lead to death is still unknown, but acute renal failure and acute respiratory distress syndrome might act as secondary contributing factors to death [51]. People who previously acquired *P*. *falciparum* malaria and those staying in malaria-endemic regions are known to show less severe symptoms during subsequent malaria infections than are people who are from non-malaria endemic regions. A previous study investigated two cases of *P*. *ovale* in patients, and it was suggested that one patient with a history of malaria was protected against developing severe complications, while the other patient, who was experiencing malaria infection for the first time, suffered severe complications [51]. Therefore, severe *P*. *ovale* infections can occur in nonimmune individuals in particular.

This systematic review indicated that jaundice, severe anemia, and pulmonary impairment are the severe complications most commonly found in patients infected with *P*. *ovale*. For jaundice, a previous study indicated that mild hyperbilirubinemia can be found in approximately 50% of patients infected with *P*. *ovale* [52]. Jaundice concomitant with *P*. *ovale* infection might be due to liver dysfunction to conjugate bilirubin in cases of severe red blood cell destruction during malaria infection. Severe anemia concomitant with *P*. *ovale* infection might occur in patients with underlying diseases such as hemoglobinopathies, causing the severe destruction of abnormal red blood cells. The mechanism of pulmonary impairment in patients with *P*. *ovale* malaria is not clear. In patients with *P*. *falciparum* infection, cytoadherence, mechanical obstruction, and inflammation of the microvascular endothelium at the pulmonary vasculature due to infected red blood cells that cytoadhere to the microvascular endothelium causes the mechanical obstruction of pulmonary vasculature; thus, alveolar capillary permeability increases, and intravascular fluid is spread into the lungs, leading to pulmonary failure [53].

### Summary of the evidence

The present systematic review and meta-analysis presented cases of severe manifestations and demonstrated the severity and mortality of severe *P*. *ovale* infection to increase the awareness of physicians regarding the prognosis of this disease. It is of the utmost importance to test individuals for malaria after they return from malaria-endemic areas, as severe *P*. *ovale* complications could occur, especially in nonimmune travelers. Last, the knowledge on appropriate management strategies and the necessary interventions concerning malaria diagnosis affects the severity of malaria manifestations and patient outcomes. Clinicians’ and technicians’ familiarity with the endemicity of malaria in an area or a region therefore plays an important role in the initial stages of laboratory assessments and subsequent diagnoses.

### Limitations

A limitation of this systematic review and meta-analysis was that there were a limited number of available publications on severe complications and death related to *P*. *ovale* infection. Another limitation was that results from case series and case-control studies were used for prevalence estimations. However, previous studies XXX have suggested and used case reports/series in meta-analyses to facilitate the decision making process when the evidence was limited or a relatively rare or neglected disease was assessed [54–59]. In addition, the most recently published meta-analysis of 44 case series reported the 25-year pooled survival of hip replacements [60]. Considering these limitations, the meta-analysis results and conclusions on the prevalence of severity and mortality associated with *P*. *ovale* infection should be considered with caution in combination with the results of newly published reports.

## Conclusion

This systematic review demonstrated that although *P*. *ovale* infections have long been considered cases of benign malaria, severe complications in patients have been reported on rare occasions. The possible reasons for the small number of reports on the severity of the malaria infections linked or caused directly by *P*. *ovale* should be studied further by the scientific community. Clinicians and technicians need to recognize that patients who return from malaria-endemic areas may develop severe *P*. *ovale* infections. Additional studies need to consider the potential underreported presence of *P*. *ovale* malaria infections in the clinical setting so that appropriate management strategies and interventions can be administered to infected individuals and the knowledge on malaria epidemiology can be expanded further.

## Supporting information

S1 Checklist PRISMA checklist. PRISMA statement for reporting systematic reviews and meta-analyses.
(DOC)

S1 Table Search term. (DOCX)

## References

[1] World Health Organization. Guidelines for the treatment of malaria. 3^rd^ ed. Geneva: WHO; 2015.

[2] Stephens JWW. A new malaria parasite of man. Ann Trop Med Parasitol. 1922;16:383–8.

[3] Collins WE, Jeffery GM. Plasmodium ovale: parasite and disease. Clin Microbiol Rev. 2005;18(3):570–81. 10.1128/CMR.18.3.570-581.2005 16020691 PMC1195966

[4] Mehlotra RK, Lorry K, Kastens W, Miller SM, Alpers MP, Bockarie M, et al Random distribution of mixed species malaria infections in Papua New Guinea. The American journal of tropical medicine and hygiene. 2000;62(2):225–31. 10.4269/ajtmh.2000.62.225 10813477

[5] May J, Mockenhaupt FP, Ademowo OG, Falusi AG, Olumese PE, Bienzle U, et al High rate of mixed and subpatent malarial infections in southwest Nigeria. The American journal of tropical medicine and hygiene. 1999;61(2):339–43. 10.4269/ajtmh.1999.61.339 10463691

[6] Dini S, Douglas NM, Poespoprodjo JR, Kenangalem E, Sugiarto P, Plumb ID, et al The risk of morbidity and mortality following recurrent malaria in Papua, Indonesia: a retrospective cohort study. BMC Med. 2020;18(1):28 10.1186/s12916-020-1497-0 32075649 PMC7031957

[7] Murphy KJ, Conroy AL, Ddungu H, Shrestha R, Kyeyune-Byabazaire D, Petersen MR, et al Malaria parasitemia among blood donors in Uganda. Transfusion. 2020. 10.1111/trf.15775 PMC7908807 32282944

[8] Yu T, Fu Y, Kong X, Liu X, Yan G, Wang Y. Epidemiological characteristics of imported malaria in Shandong Province, China, from 2012 to 2017. Sci Rep. 2020;10(1):7568 10.1038/s41598-020-64593-1 32371895 PMC7200687

[9] Lysenko AJ, Beljaev AE. An analysis of the geographical distribution of Plasmodium ovale. Bull World Health Organ. 1969;40(3):383–94. 5306622 PMC2554635

[10] Smith AD, Bradley DJ, Smith V, Blaze M, Behrens RH, Chiodini PL, et al Imported malaria and high risk groups: observational study using UK surveillance data 1987–2006. BMJ (Clinical research ed). 2008;337:a120. 10.1136/bmj.a120 PMC2453297 18599471

[11] Tanizaki R, Kato Y, Iwagami M, Kutsuna S, Ujiie M, Takeshita N, et al Performance of rapid diagnostic tests for Plasmodium ovale malaria in Japanese travellers. Trop Med Health. 2014;42(4):149–53. 10.2149/tmh.2014-07 25473374 PMC4229013

[12] Moody A. Rapid diagnostic tests for malaria parasites. Clin Microbiol Rev. 2002;15(1):66–78. 10.1128/cmr.15.1.66-78.2002 11781267 PMC118060

[13] McMorrow ML, Aidoo M, Kachur SP. Malaria rapid diagnostic tests in elimination settings—can they find the last parasite? Clin Microbiol Infect. 2011;17(11):1624–31. 10.1111/j.1469-0691.2011.03639.x 21910780 PMC4821879

[14] Mason DP, Kawamoto F, Lin K, Laoboonchai A, Wongsrichanalai C. A comparison of two rapid field immunochromatographic tests to expert microscopy in the diagnosis of malaria. Acta Trop. 2002;82(1):51–9. 10.1016/s0001-706x(02)00031-1 11904103

[15] Snounou G, Viriyakosol S, Jarra W, Thaithong S, Brown KN. Identification of the four human malaria parasite species in field samples by the polymerase chain reaction and detection of a high prevalence of mixed infections. Mol Biochem Parasitol. 1993;58(2):283–92. 10.1016/0166-6851(93)90050-8 8479452

[16] Sharma S, Kaitholia K, Bharti RS, Singh MP, Mishra N. Novel molecular diagnostic technique for detecting the different species of Plasmodium. Infect Genet Evol. 2020;78:104122 10.1016/j.meegid.2019.104122 31751755

[17] Sutherland CJ, Tanomsing N, Nolder D, Oguike M, Jennison C, Pukrittayakamee S, et al Two nonrecombining sympatric forms of the human malaria parasite Plasmodium ovale occur globally. J Infect Dis. 2010;201(10):1544–50. 10.1086/652240 20380562

[18] WHO. World malaria report 2018. https://www.who.int/malaria/publications/world-malaria-report-2018/en/

[19] Hwang J, Cullen KA, Kachur SP, Arguin PM, Baird JK. Severe morbidity and mortality risk from malaria in the United States, 1985–2011. Open Forum Infect Dis. 2014;1(1):ofu034 10.1093/ofid/ofu034 25734104 PMC4324198

[20] Lau YL, Lee WC, Tan LH, Kamarulzaman A, Syed Omar SF, Fong MY, et al Acute respiratory distress syndrome and acute renal failure from Plasmodium ovale infection with fatal outcome. Malaria journal. 2013;12:389 10.1186/1475-2875-12-389 24180319 PMC4228392

[21] D'Abramo A, Gebremeskel Tekle S, Iannetta M, Scorzolini L, Oliva A, Paglia MG, et al Severe Plasmodium ovale malaria complicated by acute respiratory distress syndrome in a young Caucasian man. Malar J. 2018;17(1):139 10.1186/s12936-018-2289-2 29609605 PMC5879577

[22] Hachimi MA, Hatim EA, Moudden MK, Elkartouti A, Errami M, Louzi L, et al The acute respiratory distress syndrome in malaria: Is it always the prerogative of Plasmodium falciparum? Rev Pneumol Clin. 2013;69(5):283–6. 10.1016/j.pneumo.2013.03.001 23688721

[23] Haydoura S, Mazboudi O, Charafeddine K, Bouakl I, Baban TA, Taher AT, et al Transfusion-related Plasmodium ovale malaria complicated by acute respiratory distress syndrome (ARDS) in a non-endemic country. Parasitol Int. 2011;60(1):114–6. 10.1016/j.parint.2010.10.005 20971212

[24] Lau YL, Lee WC, Tan LH, Kamarulzaman A, Omar SFS, Fong MY, et al Acute respiratory distress syndrome and acute renal failure from Plasmodium ovale infection with fatal outcome. Malar J. 2013;12:8 10.1186/1475-2875-12-8 24180319 PMC4228392

[25] Rojo-Marcos G, Cuadros-Gonzalez J, Mesa-Latorre JM, Culebras-Lopez AM, de Pablo-Sanchez R. Acute respiratory distress syndrome in a case of Plasmodium ovale malaria. The American journal of tropical medicine and hygiene. 2008;79(3):391–3. 18784231

[26] Strydom KA, Ismail F, Frean J. Plasmodium ovale: A case of not-so-benign tertian malaria. Malar J. 2014;13(1):85. 24612535 10.1186/1475-2875-13-85 PMC3984724

[27] Tomar LR, Giri S, Bauddh NK, Jhamb R. Complicated malaria: A rare presentation of Plasmodium ovale. Trop Doct. 2015;45(2):140–2. 10.1177/0049475515571989 25672340

[28] Wangdahl A, Wyss K, Saduddin D, Bottai M, Ydring E, Vikerfors T, et al Severity of Plasmodium falciparum and non-falciparum malaria in travelers and migrants: A nationwide observational study over 2 decades in Sweden. J Infect Dis. 2019;220(8):1335–45. 10.1093/infdis/jiz292 31175365 PMC6743839

[29] Groger M, Fischer HS, Veletzky L, Lalremruata A, Ramharter M. A systematic review of the clinical presentation, treatment and relapse characteristics of human Plasmodium ovale malaria. Malaria journal. 2017;16(1):112 10.1186/s12936-017-1759-2 28284211 PMC5346189

[30] Yerlikaya S, Campillo A, Gonzalez IJ. A systematic review: Performance of rapid diagnostic tests for the detection of Plasmodium knowlesi, Plasmodium malariae, and Plasmodium ovale monoinfections in human blood. J Infect Dis. 2018;218(2):265–76. 10.1093/infdis/jiy150 29554284 PMC6009649

[31] Lo CK, Mertz D, Loeb M. Newcastle-Ottawa Scale: comparing reviewers' to authors' assessments. BMC Med Res Methodol. 2014;14:45 10.1186/1471-2288-14-45 24690082 PMC4021422

[32] Bottieau E, Clerinx J, Van Den Enden E, Van Esbroeck M, Colebunders R, Van Gompel A, et al Imported non-Plasmodium falciparum malaria: A five-year prospective study in a European referral center. Am J Trop Med Hyg. 2006;75(1):133–8. 16837719

[33] de Laval F, Oliver M, Rapp C, Pommier de Santi V, Mendibil A, Deparis X, et al The challenge of diagnosing Plasmodium ovale malaria in travellers: report of six clustered cases in French soldiers returning from West Africa. Malaria journal. 2010;9:358 10.1186/1475-2875-9-358 21143962 PMC3017537

[34] Langford S, Douglas NM, Lampah DA, Simpson JA, Kenangalem E, Sugiarto P, et al Plasmodium malariae infection associated with a high burden of anemia: A hospital-based surveillance study. PLoS Negl Trop Dis. 2015;9(12):e0004195 10.1371/journal.pntd.0004195 26720002 PMC4697806

[35] Ramos JM, Tissiano G, Gosa A, Alegria I, Berzosa P. Study of the prevalence of Plasmodium infections by the polymerase chain reaction (PCR) among patients with severe anaemia treated at a rural hospital in southern Ethiopia. Bol Malariol Salud Ambient. 2016;56(1):75–7.

[36] Rojo-Marcos G, Rubio-Munoz JM, Angheben A, Jaureguiberry S, Garcia-Bujalance S, Tomasoni LR, et al Prospective comparative multi-centre study on imported Plasmodium ovale wallikeri and Plasmodium ovale curtisi infections. Malar J. 2018;17:11 10.1186/s12936-017-2153-9 30376868 PMC6208040

[37] Rojo-Marcos G, Rubio-Muñoz JM, Ramírez-Olivencia G, García-Bujalance S, Elcuaz-Romano R, Díaz-Menéndez M, et al Comparison of imported Plasmodium ovale curtisi and P. ovale wallikeri infections among patients in Spain, 2005–2011. Emerg Infect Dis. 2014;20(3):409–16. 10.3201/eid2003.130745 24572501 PMC3944870

[38] Wångdahl A, Wyss K, Saduddin D, Bottai M, Ydring E, Vikerfors T, et al Severity of Plasmodium falciparum and non-falciparum malaria in travelers and migrants: A Nationwide Observational Study over 2 Decades in Sweden. J Infect Dis. 2019;220(8):1335–45. 10.1093/infdis/jiz292 31175365 PMC6743839

[39] Cochrane. Cochrane Handbook for Systematic Reviews of Interventions version 6.0 (updated July 2019). 2019 [Available from: https://training.cochrane.org/handbook/current.

[40] Faye FB, Spiegel A, Tall A, Sokhna C, Fontenille D, Rogier C, et al Diagnostic criteria and risk factors for Plasmodium ovale malaria. J Infect Dis. 2002;186(5):690–5. 10.1086/342395 12195357

[41] Mueller I, Zimmerman PA, Reeder JC. Plasmodium malariae and Plasmodium ovale—the "bashful" malaria parasites. Trends Parasitol. 2007;23(6):278–83. 10.1016/j.pt.2007.04.009 17459775 PMC3728836

[42] Obare P, Ogutu B, Adams M, Odera JS, Lilley K, Dosoo D, et al Misclassification of Plasmodium infections by conventional microscopy and the impact of remedial training on the proficiency of laboratory technicians in species identification. Malaria journal. 2013;12:113 10.1186/1475-2875-12-113 23537145 PMC3626703

[43] Calderaro A, Piccolo G, Perandin F, Gorrini C, Peruzzi S, Zuelli C, et al Genetic polymorphisms influence Plasmodium ovale PCR detection accuracy. J Clin Microbiol. 2007;45(5):1624–7. 10.1128/JCM.02316-06 17360843 PMC1865880

[44] Leder K, Black J, O'Brien D, Greenwood Z, Kain KC, Schwartz E, et al Malaria in travelers: a review of the GeoSentinel surveillance network. Clin Infect Dis. 2004;39(8):1104–12. 10.1086/424510 15486832

[45] Milne LM, Kyi MS, Chiodini PL, Warhurst DC. Accuracy of routine laboratory diagnosis of malaria in the United Kingdom. J Clin Pathol. 1994;47(8):740–2. 10.1136/jcp.47.8.740 7962629 PMC502149

[46] Bigaillon C, Fontan E, Cavallo JD, Hernandez E, Spiegel A. Ineffectiveness of the Binax NOW malaria test for diagnosis of Plasmodium ovale malaria. J Clin Microbiol. 2005;43(2):1011 10.1128/JCM.43.2.1011.2005 15695736 PMC548116

[47] Marx A, Pewsner D, Egger M, Nuesch R, Bucher HC, Genton B, et al Meta-analysis: accuracy of rapid tests for malaria in travelers returning from endemic areas. Ann Intern Med. 2005;142(10):836–46. 10.7326/0003-4819-142-10-200505170-00009 15897534

[48] Brent AJ, Angus BJ. Not all that is malaria is falciparum. Lancet Infect Dis. 2008;8(3):208 10.1016/S1473-3099(08)70044-6 18291342

[49] Imbert P, Rapp C, Buffet PA. Pathological rupture of the spleen in malaria: Analysis of 55 cases (1958–2008). Travel Med Infect Dis. 2009;7(3):147–59. 10.1016/j.tmaid.2009.01.002 19411041

[50] Nolder D, Oguike MC, Maxwell-Scott H, Niyazi HA, Smith V, Chiodini PL, et al An observational study of malaria in British travellers: Plasmodium ovale wallikeri and Plasmodium ovale curtisi differ significantly in the duration of latency. BMJ Open. 2013;3(5): e002711 10.1136/bmjopen-2013-002711 23793668 PMC3657643

[51] Lau YL, Lee WC, Tan LH, Kamarulzaman A, Syed Omar SF, Fong MY, et al Acute respiratory distress syndrome and acute renal failure from Plasmodium ovale infection with fatal outcome. Malar J. 2013;12(1):389. 24180319 10.1186/1475-2875-12-389 PMC4228392

[52] Taylor T, Agbenyega T. Malaria. In: Magill AJ, Hill DR, Solomon T, Ryan ET, editors. Hunter's Tropical Medicine and Emerging Infectious Disease. 9^th^ ed. Saunders: Elsevier B.V.; 2012.

[53] Taylor WR, White NJ. Malaria and the lung. Clin Chest Med. 2002;23(2):457–68. 10.1016/s0272-5231(02)00004-7 12092039

[54] Murad MH, Sultan S, Haffar S, Bazerbachi F. Methodological quality and synthesis of case series and case reports. BMJ Evid Based Med. 2018;23(2):60–3. 10.1136/bmjebm-2017-110853 29420178 PMC6234235

[55] Nissen T, Wynn R. The clinical case report: a review of its merits and limitations. BMC Res Notes. 2014;7:264 10.1186/1756-0500-7-264 24758689 PMC4001358

[56] Nakamura T, Igarashi H, Ito T, Jensen RT. Important of case-reports/series, in rare diseases: Using neuroendocrine tumors as an example. World J Clin Cases. 2014;2(11):608–13. 10.12998/wjcc.v2.i11.608 25405184 PMC4233424

[57] Jackson D, Daly J, Saltman DC. Aggregating case reports: a way for the future of evidence-based health care? Clin Case Rep. 2014;2(2):23–4. 10.1002/ccr3.58 25356237 PMC4184623

[58] Sampayo-Cordero M, Miguel-Huguet B, Pardo-Mateos A, Malfettone A, Perez-Garcia J, Llombart-Cussac A, et al Agreement between results of meta-analyses from case reports and clinical studies, regarding efficacy and safety of idursulfase therapy in patients with mucopolysaccharidosis type II (MPS-II). A new tool for evidence-based medicine in rare diseases. Orphanet J Rare Dis. 2019;14(1):230 10.1186/s13023-019-1202-6 31639024 PMC6805333

[59] Chambers D, Rodgers M, Woolacott N. Not only randomized controlled trials, but also case series should be considered in systematic reviews of rapidly developing technologies. J Clin Epidemiol. 2009;62(12):1253–60 e4. 10.1016/j.jclinepi.2008.12.010 19349144

[60] Evans JT, Evans JP, Walker RW, Blom AW, Whitehouse MR, Sayers A. How long does a hip replacement last? A systematic review and meta-analysis of case series and national registry reports with more than 15 years of follow-up. Lancet. 2019;393(10172):647–54. 10.1016/S0140-6736(18)31665-9 30782340 PMC6376618

