# Supplementary material for: Severity and mortality of severe Plasmodium ovale infection: A systematic review and meta-analysis
Source: PLoS One. 2020 Jun 19;15(6):e0235014. doi: 10.1371/journal.pone.0235014 (PMC7304606; doi:10.1371/journal.pone.0235014)
Supplement: S1 Table — (DOCX) [file pone.0235014.s002.docx]

**Severity and mortality of severe *Plasmodium ovale* infection: a systematic review and meta-analysis**

Manas Kotepui^1*^, Kwuntida Uthaisar Kotepui^1^, Giovanni D Milanez^2^, Frederick R Masangkay^2^

^1^Medical Technology, School of Allied Health Sciences, Walailak University, Thasala, Nakhon Si Thammarat, Thailand

^2^Department of Medical Technology, Institute of Arts and Sciences, Far Eastern University-Manila, Manila, Philippines

Authors’ Email Address:

**^*^Corresponding Author**: Manas Kotepui; manas.ko@wu.ac.th, +66954392469

Kwuntida Uthaisar Kotepui; kwuntida.ut@wu.ac.th

Giovanni D Milanez; gmilanez@feu.edu.ph

Frederick R Masangkay; frederick_masangkay2002@yahoo.com

**Table S1 Search term**

| **Databases** | **Search terms** | **Date** |
| --- | --- | --- |
| PubMed | (severe OR complicated OR Complication) AND (Plasmodium ovale) | 5 January 2020 |
| Scopus | (severe OR complicated OR Complication) AND (Plasmodium ovale)  Search option: All fields | 5 January 2020 |
| ISI Web of Science | (severe OR complicated OR Complication) AND (Plasmodium ovale)  Search option: All fields | 5 January 2020 |
